# A field boundary dataset for the canadian prairies derived from sentinel-2 imagery using the segment anything model

**DOI:** 10.1016/j.dib.2026.112691

**Published:** 2026-03-23

**Authors:** Thuan Ha, Kwabena Abrefa Nketia, Shawn Neudorf, Steve J. Shirtliffe

**Affiliations:** Department of Plant Science, College of Agriculture and Bioresources, University of Saskatchewan, Saskatoon, SK S7N 5A8, Canada

**Keywords:** Agriculture, Field border, Remote sensing, Segmentation, Geo-informatics

## Abstract

This article presents a Prairie-wide spatial vector dataset of agricultural field boundaries across Alberta, Saskatchewan, and Manitoba, Canada. The dataset was generated from Sentinel-2 Level-2A surface reflectance imagery (10 m spatial resolution) using an automated segmentation workflow based on the Segment Anything Model version 2 (SAM2). Sentinel-2 imagery was accessed in Google Earth Engine (GEE), filtered using cloud/quality masks, and aggregated into seasonal RGB composites representing key crop phenological periods (early-, mid-, and late-season) for large-scale segmentation input. The composite images were exported and processed with a SAM2 segmentation pipeline (tiled inference and mosaic-based post-processing) to delineate candidate field units without manually labeled training samples. Segmentation outputs were then post-processed using rule-based filtering and topology repair (removal of small artifacts/sliver polygons, hole filling, boundary cleaning, and geometry validity correction). Final vector outputs are distributed in ESRI Shapefile and GeoParquet formats with geometry attributes for downstream spatial analysis. The workflow code and processing scripts are provided to support reproducibility and adaptation to other regions. This dataset provides a consistent field-scale boundary reference layer for agricultural monitoring, crop and yield modeling, soil and environmental analysis, cropland mapping, land management, and machine-learning applications across the Canadian Prairies.

• Prairie-wide field boundary datasets generated using automated segmentation of seasonal Sentinel-2 composites with the Segment Anything Model version 2, followed by vectorization and topological post-processing.
• Vector outputs provided in both Shapefile and GeoParquet formats, enabling efficient use in traditional GIS, cloud-native, and big-data geospatial workflows.
• A consistent, large-scale field boundary reference dataset supporting agricultural analysis, spatial modeling, cropland mapping, and machine learning applications across the Canadian Prairies.

Specifications Table

**Table:** 

Subject	Earth & Environmental Sciences
Specific subject area	Agriculture Science, Remote Sensing
Type of data	Geospatial polygon data in shapefile and GeoParquet format
Data collection	Sentinel-2 imagery was collected for the Canadian Prairies for the 2021–2024 growing seasons (May–September) using GEE. To organize downloads and processing, cropland areas were divided into tile units based on census subdivision polygons. For each tile and year, Sentinel-2 scenes were filtered to retain images with <50 % cloud cover, then clipped, temporally aggregated, and masked. Annual seasonal RGB composites were generated by median compositing with predefined date windows (Red: May–July, Green: July–August, Blue: August–September). A 10 m ESA WorldCover-derived cropland mask was applied to exclude non-cropland pixels before the composite mosaics were exported for subsequent segmentation and field-boundary extraction. The final output dataset is a vector field-boundary map of agricultural areas across the Canadian Prairies.
Data source location	The dataset covers major agricultural regions across the Canadian Prairies, spanning approximately 49°–55° N latitude and 96°–114° W longitude. The processed dataset is stored and maintained by the authors’ institution.
Data accessibility	Repository name: A field boundary dataset for the Canadian Prairies derived from Sentinel-2 imagery using the Segment Anything Model version 2Data identification number: 10.17632/2y568rt76w.1 Direct URL to data: 1. For downloading: https://data.mendeley.com/datasets/2y568rt76w/1 2. For displaying: https://soiladf2025.projects.earthengine.app/view/field-boundary-canadian-prairies 3. Link to repository: ✓ https://github.com/thuanhavan/Prairies-Field-Boundary-Data 4. Link to methods to reproduce the dataset ✓ https://github.com/thuanhavan/CSA_Field_Boundary_Segmentation This dataset provides field boundary polygons for cultivated land across the Canadian Prairies derived from Sentinel-2 imagery using the Segment Anything Model (SAM).1. Downloading the Dataset The complete dataset can be downloaded from Mendeley Data using the following link:https://data.mendeley.com/datasets/2y568rt76w/1Steps:
	• Open the download URL in a web browser. • Select the desired file(s) or download the full dataset as a ZIP archive. • Extract the downloaded files to a local directory (shp or GeoParquet). The dataset is provided in standard and cloud-optimized geospatial formats (e.g., vector polygon files, GeoParquet) and can be opened using common GIS software such as QGIS, ArcGIS Pro, or programmatically using Python (e.g., geopandas) or R (e.g., sf). 2. Visualizing the Data Online (No Download Required) An interactive web application is provided for quick visualization and exploration of the field boundaries without downloading the data:Online Viewer:https://cropagronomyusask.users.earthengine.app/view/field-boundariesSteps: • Open the viewer link in a modern web browser. • Use the map controls to zoom and pan across the Canadian Prairies. • Toggle layers and inspect field boundary polygons directly on the map. • This viewer is intended for visualization and quality inspection only; it does not replace the downloadable dataset for analysis. 3. Using the Data in GIS and Analysis WorkflowsThe field boundary polygons can be integrated into GIS workflows for spatial analysis, mapping, and overlay with other geospatial datasets (e.g., crop type maps, yield data, soil properties, or climate variables). The data are suitable for applications in precision agriculture, land-use mapping, crop monitoring, and large-scale geospatial modeling. When used in scientific publications or derivative works, please cite the dataset using the provided doi:10.17632/2y568rt76w.1
Related research article	Ha, Thuan and Nketia, Kwabena Abrefa and Fernando, Hansanee and van Steenbergen, Sarah and Neudorf, Shawn and Shirtliffe, Steve J., Field boundary delineation with seasonal Sentinel 2 imagery using Segment-Anything Model (SAM). Available at MethodsX: https://doi.org/10.1016/j.mex.2026.103794

## 1 Value of the Data

• Provides a large-scale, spatially consistent vector representation of agricultural field boundaries across the Canadian Prairies, enabling standardized field-level spatial units for agricultural and environmental analyses.
• Supplies reusable field boundary datasets that can serve as spatial reference layers for crop monitoring, yield modeling, soil property mapping, and land management studies requiring consistent delineation of individual agricultural fields.
• Supports the development, training, and validation of machine learning and deep learning models for agricultural mapping, image segmentation, zonal statistics, and field-scale classification using remote sensing data.
• Enables regional- to national-scale studies that require reproducible and consistent field boundary delineation across heterogeneous Prairie agricultural landscapes.

## 2 Background

The segmented field boundary dataset was compiled to provide a reusable and standardized spatial representation of agricultural field boundaries across the Canadian Prairies. Consistent field-scale spatial units are a foundational requirement for many downstream geospatial analyses, including zonal statistics for crop yield prediction in precision agriculture, greenhouse gas emission risk assessment, and marginal land mapping across Prairie agricultural landscapes. By providing processed field boundary datasets, this resource enables users to directly access delineated field geometries without the need to acquire satellite imagery or re-run computationally intensive segmentation workflows repeatedly. The data were generated using a fully automated field boundary delineation workflow that integrates time-series Sentinel-2 imagery and the Segment Anything Model (SAM) version 2 [1], a pre-trained foundation vision model for image segmentation. Seasonal Red-Green-Blue composite images were produced at key crop phenological stages using Sentinel-2 surface reflectance data [2] accessed through Google Earth Engine (GEE). These composites were used as inputs to SAM to generate raster segmentation masks, which were subsequently post-processed to remove artifacts, enforce spatio-temporal consistency, and converted into vector polygon field boundaries. This Data in Brief article is associated with a related MethodsX manuscript [3] that documents the segmentation workflow, while the present article focuses on describing and disseminating the resulting spatial dataset for reuse in agricultural, environmental, and geospatial research applications.

## 3 Data Description

The dataset comprises vector polygon files representing agricultural field boundaries across the Canadian Prairies, covering three provinces: Alberta, Saskatchewan, and Manitoba. Field boundaries were automatically delineated through image segmentation algorithms applied to satellite imagery, resulting in a comprehensive and spatially consistent inventory of field geometries across the region.

The dataset includes field boundary data for 457 Rural Municipality (RM) divisions and contains a total of 656,082 individual field polygons representing approximately 27.5 million hectares of agricultural land. Each polygon represents a distinct agricultural field and includes geometric and administrative attributes suitable for field identification, area, and perimeter measurements. The dataset is distributed as ESRI Shapefiles (.shp, .shx, .dbf, .prj, .cpg) and GeoParquet (.parquet) formats projected to the World Geodetic System 1984 (WGS 84; EPSG:4326) geographic coordinate system, ensuring compatibility with standard GIS software and seamless integration with other regional and national datasets.

Attribute fields include field_id (unique identifier); area_ha (field area in hectares); border_len (perimeter length in meters); RM_number (Rural Municipality code); and RM_name (Rural Municipality name). Attribute definitions and naming conventions are standardized across all provincial datasets to facilitate consistent regional analysis and cross-jurisdictional comparisons.

The dataset is organized by province and Rural Municipality, with coverage comprising 66 RMs in Alberta: 225,784 field polygons, 295 RMs, in Saskatchewan: 315,503 field polygons and 96 RMs in Manitoba: 114,795 field polygons. Supplementary metadata files accompany each RM division and document spatial reference information, attribute definitions, and file-level metadata.

The novelty of this dataset lies in the preprocessing strategy used to generate segmentation-ready inputs before applying the foundation model. Rather than using a single-date image, we compiled seasonally structured RGB composites from Sentinel-2 time series (with band assignment based on different phenological windows), which enhances field-edge contrast and improves the visibility of field patterns across diverse crop conditions. In addition, a cropland mask was applied prior to segmentation to remove non-agricultural areas from the input imagery, thereby reducing irrelevant objects, improving segmentation efficiency, and increasing the consistency of extracted field boundaries. This combination of phenology-informed RGB compositing and pre-segmentation cropland masking is a key methodological feature that enabled large-scale, automated field-boundary extraction across the Canadian Prairies.

## 4 Experimental Design, Materials and Methods

### 4.1 Data location

**Fig. 1:**
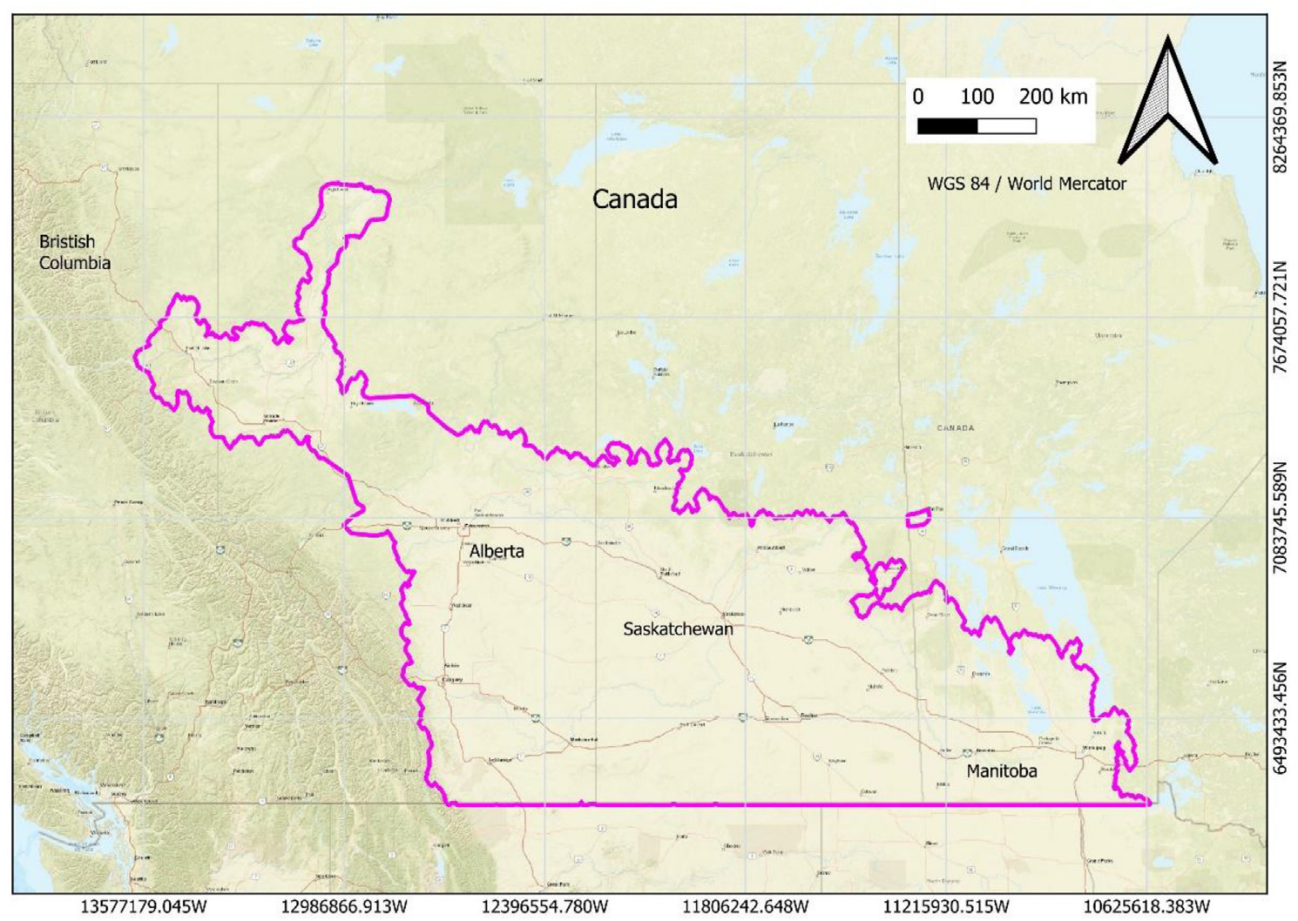
Spatial extent of the dataset showing agricultural regions of the Canadian Prairies, including Alberta, Saskatchewan, Manitoba, and part of British Columbia.

The dataset covers agricultural regions of the Canadian Prairies, including Alberta, Saskatchewan, Manitoba, and portions of British Columbia. The spatial extent of the study area spans approximately 49° to 55° N latitude and 96° to 114° W longitude (Fig. 1). The processed field boundary datasets are stored and maintained at the authors’ institutional affiliation and are publicly available through the associated data repository accompanying this article.

### 4.2 Data sources

**Fig. 2:**
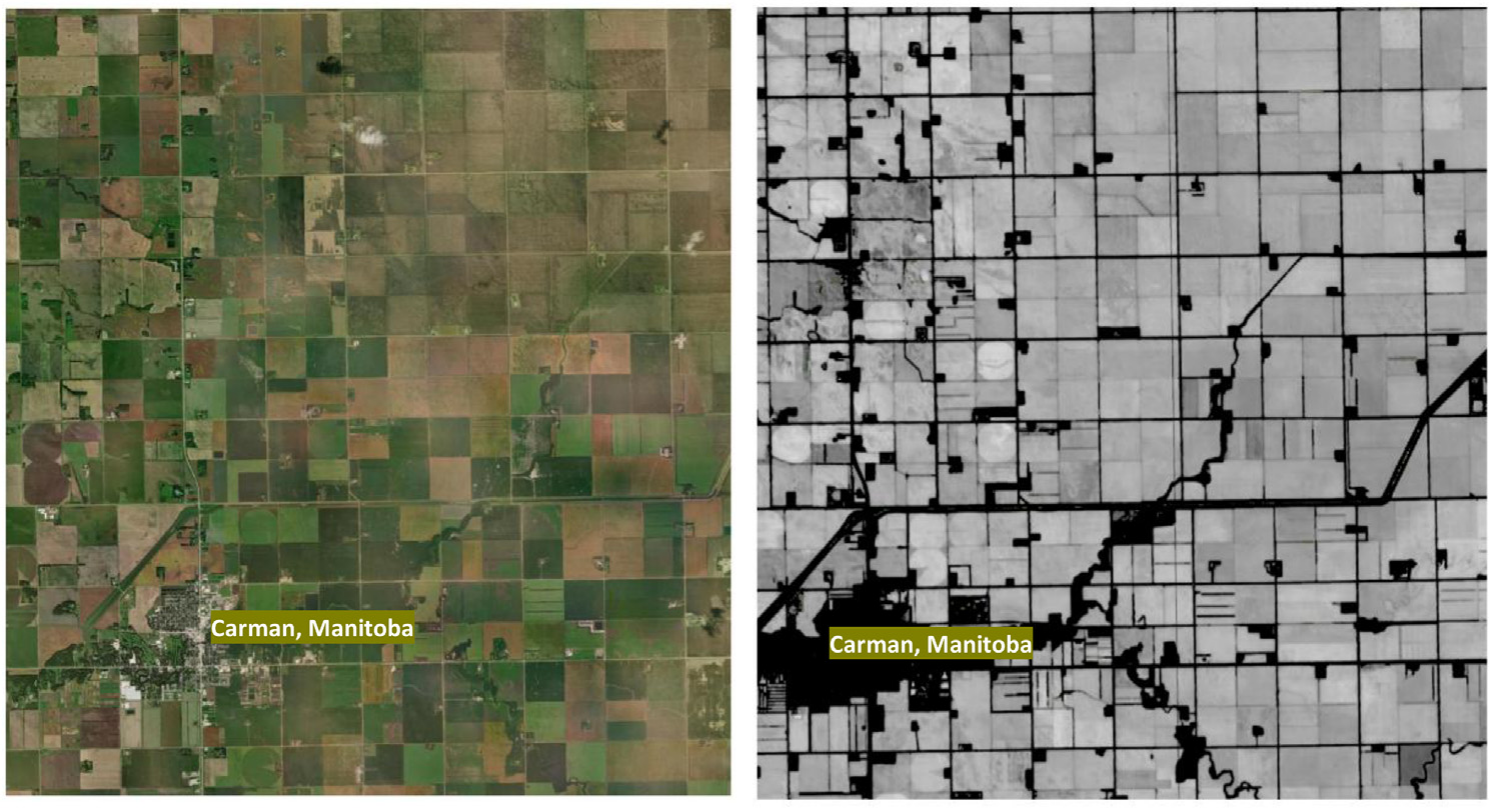
Examples of a base map (left) and crop mask (right) in Google Earth Engine (GEE).

Multispectral Sentinel-2 surface reflectance imagery was used as the primary data source for field boundary delineation. Sentinel-2 images were accessed through GEE [2] and filtered to include observations covering the Canadian Prairies (Alberta, Saskatchewan, Manitoba, and part of British Columbia). Image acquisition dates were selected to represent the main agricultural growing season (May to September), and image scenes were screened for cloud contamination using standard quality assurance bands and cloud-masking procedures available within GEE. To restrict the analysis to agricultural areas, an Agriculture and Agri-Food Canada (AAFC) annual crop mask [4] was applied before segmentation to exclude non-agricultural land cover. Fig. 2 shows an example of the base Sentinel-2 imagery and the applied crop mask for a representative area in Manitoba.

### 4.3 Seasonal composite generation

**Fig. 3:**
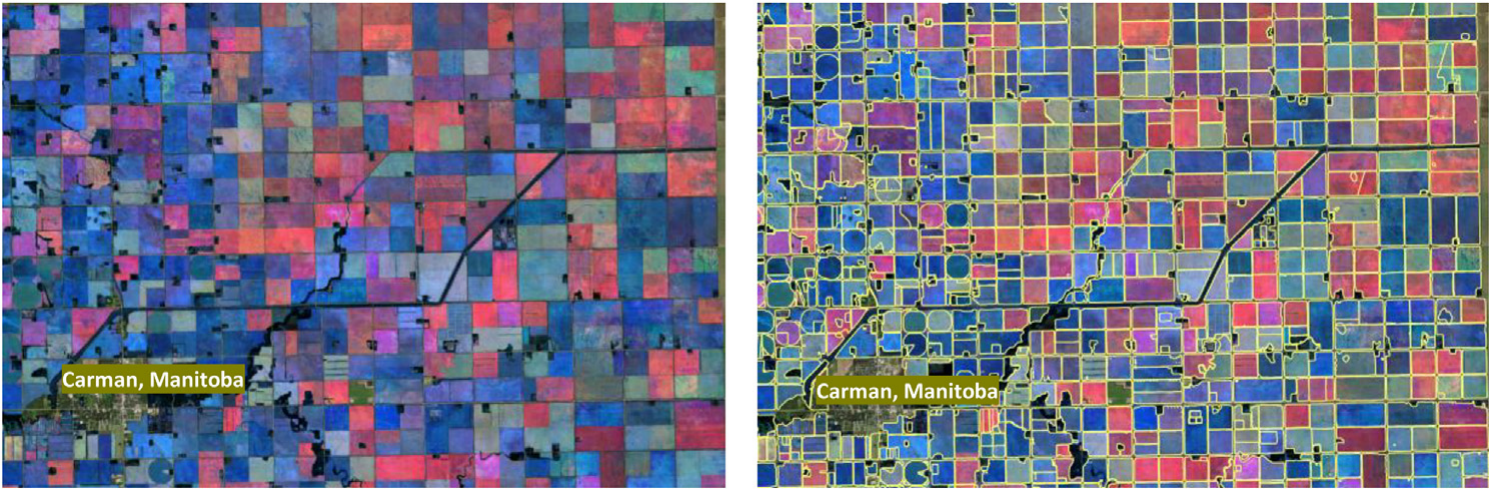
Examples of seasonal RGB (left) and field boundary map layer (right).

Sentinel-2 imagery acquired during the 2021–2024 growing season was grouped into seasonal periods corresponding to key crop phenological stages. For each seasonal period, the Red, Green, and Blue (RGB) bands were composited using median pixel values to reduce residual cloud effects, atmospheric variability, and temporal noise. The AAFC cropland mask was then applied to each seasonal RGB composite to retain cropland pixels and exclude non-agricultural areas, resulting in cropland-only RGB images (Fig. 3). These masked seasonal RGB composites served as inputs for subsequent image segmentation and field boundary extraction workflows.

### 4.4 Field boundary segmentation

Masked seasonal RGB composites (Fig. 3) were used as inputs to the Segment Anything Model (SAM) version 2, a foundation vision model developed by Meta AI for general-purpose image segmentation [1]. SAM was applied in a fully automated workflow without manually labeled training data or user-defined prompts. Segmentation was performed at full Sentinel-2 spatial resolution to generate raster masks representing potential agricultural field boundaries across the study area.

### 4.5 Post-processing and vectorization

Segmentation outputs were post-processed to improve spatial accuracy and remove artifacts. Raster masks were filtered to remove non-cropland artifacts and small isolated regions based on area thresholds. The cleaned raster masks were then converted into vector polygon features using geospatial raster-to-vector conversion tools. Polygon geometries were further processed in the ArcPy environment (Esri. ArcGIS Pro version 3.5, Redlands, CA, USA) to enforce topological consistency. Post-processing steps included removal of sliver polygons, geometry simplification, and boundary smoothing where required, which resulted in contiguous and clean vector representations of agricultural fields suitable for spatial analysis and integration with other geospatial datasets.

### 4.6 Dataset assembly

Final field boundary polygons were saved in ESRI Shapefile format, including associated geometry files and projection information. Attribute tables were standardized across all shapefiles to include unique field identifiers and basic geometric properties (e.g., area and perimeter). In addition to Shapefiles, vector data were exported in GeoParquet format to support cloud-native, scalable geospatial workflows. All files were organized into a structured repository with accompanying metadata and documentation, facilitating reuse, integration, and reproducible analysis.

### 4.7 Software and tools

The data were generated using a reproducible computational workflow that combines cloud-based satellite data processing, automated image segmentation, and geospatial post-processing. Data access, preprocessing, and compositing were conducted using GEE, while segmentation, vectorization, and dataset assembly were performed using Python-based geospatial tools. The key software tools, libraries, and code resources used to generate the dataset are summarized below:

• **Google Earth Engine (GEE):** Cloud-based access, preprocessing, and compositing of Sentinel-2 imagery; development of an interactive web application for data visualization and exploration (GEE App: link)
• **Geospatial libraries:** Raster and vector processing, including GDAL and GeoPandas, and related tools for segmentation output handling and dataset assembly.
• **Code availability**: All scripts used for image preprocessing, segmentation execution, post-processing, dataset assembly, and GEE App development are publicly available in the associated GitHub repository:
  https://github.com/acilabuofs/field_border_segmentation

This workflow ensures transparency, reproducibility, and adaptability for future research and applications, supporting both desktop GIS and cloud-based geospatial analysis environments.

## Limitations

The dataset was generated using seasonal Red-Green-Blue composites derived from Sentinel-2 imagery. In some locations, persistent cloud cover and atmospheric conditions limited the availability of cloud-free observations during specific seasonal periods. Consequently, certain composites may contain residual cloud artifacts or reduced visual clarity, which can affect the precision of field boundary delineation in those areas. Variability in image availability across regions and seasons may also introduce spatial heterogeneity in data quality. No supplementary ground-based observations were used to compensate for areas with limited cloud-free imagery, and users should consider these factors when applying the dataset to analyses requiring high positional accuracy or complete coverage.

## Ethics Statement

None.

## CRediT Author Statement

**Thuan Ha:** Conceptualization, Methodology, Data processing, Software, Writing- Original draft preparation. **Kwabena Abrefa Nketia, Shawn Neudorf:** Software, Methodology, Validity tests, Data curation, Writing- Reviewing and Editing. **Steve J. Shirtliffe:** Supervision, Funding acquisition, Writing- Reviewing and Editing.

## Declaration of Competing Interest

The authors declare that they have no known competing financial interests or personal relationships that could have appeared to influence the work reported in this paper.

## Data Availability

Mendeley DataField boundary (Original data)

## Appendix Supplementary materials

Image, application 1
